# Local Assemblies of Paired-End Reduced Representation Libraries Sequenced with the Illumina Genome Analyzer in Maize

**DOI:** 10.1155/2012/360598

**Published:** 2012-10-09

**Authors:** Stéphane Deschamps, Kishore Nannapaneni, Yun Zhang, Kevin Hayes

**Affiliations:** ^1^DuPont Agricultural Biotechnology, P.O. Box 80353, Wilmington, DE 19880, USA; ^2^DuPont Pioneer, P.O. Box 1004, Johnston, IA 50131, USA

## Abstract

The use of next-generation DNA sequencing technologies has greatly facilitated reference-guided variant detection in complex plant genomes. However, complications may arise when regions adjacent to a read of interest are used for marker assay development, or when reference sequences are incomplete, as short reads alone may not be long enough to ascertain their uniqueness. Here, the possibility of generating longer sequences in discrete regions of the large and complex genome of maize is demonstrated, using a modified version of a paired-end RAD library construction strategy. Reads are generated from DNA fragments first digested with a methylation-sensitive restriction endonuclease, sheared, enriched with biotin and a selective PCR amplification step, and then sequenced at both ends. Sequences are locally assembled into contigs by subgrouping pairs based on the identity of the read anchored by the restriction site. This strategy applied to two maize inbred lines (B14 and B73) generated 183,609 and 129,018 contigs, respectively, out of which at least 76% were >200 bps in length. A subset of putative single nucleotide polymorphisms from contigs aligning to the B73 reference genome with at least one mismatch was resequenced, and 90% of those in B14 were confirmed, indicating that this method is a potent approach for variant detection and marker development in species with complex genomes or lacking extensive reference sequences.

## 1. Introduction

DNA-based genetic markers are pivotal tools for applications as diverse as QTL mapping, marker assisted selection, association mapping, and fine mapping for the detection of genes linked to a particular phenotype [1]. Among the variety of genetic markers that have been developed, those derived from single nucleotide polymorphisms (SNPs) have become the marker of choice for many mapping applications because of their abundance and the availability of high-throughput and cost-effective technologies for detection and diagnostics [2–4]. One popular tool for SNP identification and detection has been the construction of reduced-representation libraries (RRL) and their sequencing with massively parallel sequencing platforms, in species as varied as cattle, worm, soybean, rice, maize, or common bean [5–10]. However, one major limitation of such platforms is the relatively short length of individual sequencing reads. While the availability of a high quality reference sequence may render short reads sufficient for alignment and subsequent SNP detection, this limitation may be further compounded in crop species due to (1) the inherent complexity of genomes (and transcriptomes) in economically important species, such as maize, soybean, or canola, due to an elevation in ploidy and/or the frequent expansion of paralogous sequences and gene families, and the need to generate very long sequencing reads for resolving highly duplicated sequences within a single genome, and (2) the potentially large number of polymorphisms between lines in those same plant species (including indels and in regions flanking a polymorphism of interest) and the need to provide long line-specific sequences for identifying variants, open reading frames, or other biologically active regions for lines whose genome sequence has a significantly altered composition in comparison to the reference assembly. Because of those limitations, the Roche 454 FLX platform [11] is often used as the instrument of choice for providing long sequencing reads and generating an appropriate sequencing scaffold. However, its relatively lower sequencing throughput when compared to other second-generation sequencing instruments often means that a large number of runs are needed to generate a high quality sequencing assembly from very large genomes. More recently, the PacBio RS platform from Pacific Biosciences [12] has garnered a lot of attention due to its ability to generate very long sequencing reads, in the kilobase range, but its low raw accuracy rate makes it, for now, unpractical to use as a variant detection platform.

In a previous study [8], we developed a methodology for rapid SNP detection in rice and soybean that can be applied to a wide range of moderately or highly complex plant genomes where sufficient genomic reference sequences are available. The methodology, based on digesting the genome with a methylation-sensitive restriction endonuclease (RE) followed by a secondary digestion with the 4-bp restriction endonuclease *DpnII*, generates short DNA fragments that are sequenced at one end using the Illumina Genome Analyzer II. The resulting 32 bp sequencing reads are immediately adjacent to the *DpnII* site. Thus, they can be directly compared and aligned to a reference assembly for SNP detection. However, one problem inherent to this approach is the lack of sequencing information flanking the 32 bp read of interest and the need to rely on reference genomic sequences from a different line or species for primer design and SNP assay development. The relative short length of the sequence also can lead to relatively high false positive discovery rates in species lacking robust reference sequences.

Several studies already have shown that DNA fragments sequenced at both ends on a massively parallel sequencing platforms can be locally assembled into contigs hundreds or even thousands of bases long. Hiatt et al. [13] developed a methodology to generate long consensus sequences in *Pseudomonas aeruginosa* from short second-generation sequencing reads. In there, ~500 bps DNA fragments are sheared randomly to produce fragments shorter than the original fragment set. Those shorter fragments then are end-sequenced at both ends with the Illumina Genome Analyzer II and the resulting read pairs are grouped together and assembled to recreate the sequence of the original ~500 bp DNA fragment. More recently, Etter et al. [14] developed a paired-end restriction-site associated DNA (RAD-PE) method, where ~350–850 bps RAD fragments from three-spined sticklebacks (*Gasterosteus aculeatus*), with one end anchored by a restriction endonuclease site and the other end randomly sheared, were sequenced on the Illumina Genome Analyzer II, to locally assemble staggered randomly sheared ends associated to a particular restriction site into restriction site-specific contigs several hundred bases in length. The RAD-PE protocol requires several steps, including the digestion of genomic DNA, ligation of two independent adapters, and the selective PCR amplification of RAD fragments flanked only by the two distinct adapters. A variation in protocol, namely, partial digestion of the genomic DNA rather than full digestion, creates overlapping RAD fragments, which in turn can be assembled into overlapping contigs covering sequences several thousand bases in length. A similar concept using RAD fragments was tested by Willing et al. [15] on two guppy (*Poecilia reticulata*) populations, generating consensus ~200–400 bps sequences that were used for polymorphism discovery, checking for the presence or absence of restriction-site associated sequences in the two populations to identify them as polymorphic (as the absence of a sequence suggests the presence of a polymorphism in the restriction site).

In this study, a revised version of the RAD-PE method was tested and implemented in maize, in order to assess the feasibility of using such a strategy for SNP detection on the large and complex genome of an economically important crop. The revised RAD-PE protocol shown here extends the concept presented in Deschamps et al. [8], where SNP detection in complex crop genomes had been successfully attempted. Briefly, the digestion of DNA fragments with *DpnII *is replaced by random shearing, and the sheared DNA fragments containing the methylation-sensitive RE site at one end are recovered, size-selected, enriched, and sequenced at both ends on the Illumina Genome Analyzer IIx. Two steps are used to select for DNA fragments prior to paired-end sequencing, namely, a biotin selection step performed after the initial digestion with a methylation-sensitive RE, and a second selection step when DNA fragments are selectively amplified via PCR prior to sequencing. The resulting individual read pairs then are assembled *de novo* to create larger contigs that can be used both for SNP detection and SNP assay design for marker development. These contigs can be several hundreds bps long and their expected length can be modulated simply by selecting appropriate DNA fragment size ranges on gel. In addition to SNP discovery and assay design, they can be used in multiple species for applications whose purpose is facilitated by long sequencing reads, such as copy number variant detection, open reading frame discovery, or metagenomics. 

## 2. Materials and Methods

### 2.1. Tissue Preparation and Genomic DNA Isolation

 Maize genomic DNA samples were extracted according to the protocol described in Deschamps et al. [8].

### 2.2. Genomic DNA Preparation and Library Construction

 B73 and B14 genomic DNA (10 *μ*g) was digested for 4 h at 37°C with 15 U of *PstI* (Promega) in a total volume of 20 *μ*L of 1X *PstI* buffer (Promega) containing 1X acetylated BSA (Promega). The enzyme was inactivated at 65°C for 20 min then the digested genomic DNA was purified with QIAquick PCR purification spin columns (Qiagen). Biotinylated *PstI*-specific adapters were created by mixing 1500 pmol each of two synthetic oligonucleotides (upper strand, /5′Bio/GGTTGACATGCTGGATTGAGACCTGCAGGTGC*A, where * is a phosphorothioate bond; lower strand, /5′PO_4_-/CCTGCAGGTCTCAATCCAGCATGTC) in 100 *μ*L water, heating them up at 95°C for 2 min, and then allowing them to cool slowly to room temperature. A ~50-fold excess of *PstI*-specific adapters (37.5 pmol) relative to available *PstI* ends was ligated to the digested DNA, in a total volume of 20 *μ*L of 1X ligase buffer (New England Biolabs) containing 10,000 U of T_4_ DNA ligase (New England Biolabs). The reaction was incubated overnight at 16°C then 10 min at 70°C, and then purified with QIAquick PCR purification spin columns (Qiagen). The digested DNA was randomly sheared via nebulization at 32 psi for 6 min then purified with QIAquick PCR purification spin columns (Qiagen). 

 After random shearing, a biotin selection step allowed the capture of smaller DNA fragments containing a *PstI* site at one end. For biotin selection, 100 *μ*L of Streptavidin-Dynabeads M-280 (Invitrogen) were washed twice with 1 mL TE buffer then resuspended in 100 *μ*L 2X B&W buffer (10 mmol L^−1^ Tris-HCl, pH 7.5; 1 mmol L^−1^ EDTA; 2 mol L^−1^ NaCl). The DNA fragments then were added to the beads and incubated for 30 min at 30°C with gentle horizontal mixing. After withdrawing the supernatant, the beads were collected and washed three times with 1 mL 1X B&W buffer then three times with 1 mL TE buffer. The washed beads were resuspended in 200 *μ*L of 1X NEBuffer 4 (New England Biolabs) containing 20 U *SbfI* (New England Biolabs) and incubated for 90 min at 37°C with gentle horizontal mixing. An *SbfI* recognition site (5′-CCTGCA^*∧*^GG-3′) located immediately upstream from the *PstI* site on the biotinylated adapter allowed for the cleavage and recovery of the biotin-bound DNA fragments. The supernatant containing the released DNA fragments was then transferred to a new tube, extracted with 100 *μ*L phenol/chloroform/isoamyl alcohol (25 : 24 : 1) and precipitated with ethanol and 3 mol L^−1^ NaOAc. DNA was resuspended in 30 *μ*L EB buffer (Qiagen).

End-repair, “A” base addition and ligation of adapters were performed according to the protocol developed by Illumina for preparing DNA samples for paired-end sequencing. Buffer and enzymatic reagents were obtained directly from Illumina's PE Sample Prep Kit (catalog number PE-102-1002). Briefly, DNA was end repaired for 30 min at 20°C in a total volume of 100 *μ*L of 1X T_4_ DNA ligase buffer with 10 mM ATP containing 4 *μ*L 10 mM dNTP mix, 5 *μ*L T_4_ DNA polymerase, 1 *μ*L Klenow enzyme, and T_4_ PNK. After incubation, DNA was purified with a QIAquick PCR Purification Kit (Qiagen) and resuspended in 32 *μ*L EB buffer (Qiagen). “A” bases then were added to the 3′ ends of blunt-ended DNA fragments. DNA was incubated at 37°C for 30 min in 1X Klenow buffer containing 10 *μ*L 1 mM dATP and 3 *μ*L Klenow exo (3′ to 5′ exo minus). After incubation, DNA was purified with a QIAquick MinElute Purification Kit (Qiagen) and resuspended in 10 *μ*L EB buffer (Qiagen). Illumina PE adapters then were ligated to DNA fragments at 20°C for 15 min in a total volume of 50 *μ*L of 1X DNA ligase buffer containing 1 *μ*L PE adapter oligo mix and 5 *μ*L DNA ligase. After incubation, DNA was purified with a QIAquick PCR Purification kit (Qiagen) and resuspended in 30 *μ*L EB buffer (Qiagen). Ligated DNA then were loaded on an E-Gel 2% with SYBR Safe agarose gel (Invitrogen) in the presence of low molecular weight DNA ladder (New England Biolabs) for size selection. After running the gel for 26 min in an E-Gel PowerBase v4 electrophoresis unit (Invitrogen), a 200–500 bp DNA smear was cut out and purified with the QIAquick Gel Extraction kit (Qiagen). DNA was resuspended in 30 *μ*L EB buffer (Qiagen).

 After ligation of Illumina adapters and gel-based size selection of the 200–500 bps ligated DNA fragments, a PCR amplification step was performed using the Illumina Paired-End (PE) PCR primer 1.0 and a modified PE PCR primer 2.0 ending in 5′-TGCAGGTGCA-3′ (matching adjacent *SbfI* and *PstI* recognition sites). The use of Illumina PE adapters and of a modified PE PCR primer 2.0 further allowed the selective amplification of DNA fragments containing a *PstI* recognition site at one end. For PCR amplification, 1 *μ*L of size-selected DNA was incubated in 50 *μ*L of 1X Phusion HF master mix (Finnzymes) in the presence of PCR primer PE 1.0 (Illumina) and the modified PCR primer PE 2.0 (CAAGCAGAAGACGGCATACGAGATCGGTCTCGGCATTCCTGCTGAACCGCTCTTCCGATCTTGCAGGTGC*A, where * is a phosphorothioate bond and “TGCAGGTGC*A” is a signature 3′ sequence containing leftovers from *SbfI* & *PstI* restriction sites). After PCR amplification (30 s at 98°C, followed by 20 rounds of 10 s at 98°C, 30 s at 65°C, 30 s at 72°C, and a final extension step of 5 min at 72°C), DNA was purified with a QIAquick PCR Purification Kit (Qiagen) then subjected to a second round of gel-based size selection using conditions similar to the ones described above. The 200–500 bps amplified DNA fragments were size-selected on gel and the presence of Illumina adapters at both ends suggests that the actual sizes of the fragments that were sequenced were between ~70 bps and ~370 bps.

### 2.3. Cluster Generation and Paired-End Sequencing

 Cluster generation and paired-end sequencing were performed on an Illumina Cluster Station and Genome Analyzer IIx, respectively, according to protocols and recipes developed by Illumina. Sequencing was performed for 76 cycles at both ends of the clustered DNA fragments using PE sequencing primers for Read 1 and Read 2 (Illumina), generating read pairs, “reads 1” and “reads 2”, mapping the ends of PCR products generated with PE PCR primer 1.0 and the modified PE PCR primer 2.0, respectively. The resulting read 1 and read 2 sequences were grouped into “read pairs” according to the X and Y coordinates of the corresponding DNA cluster on the flow cell. Sequencing reads and quality scores were generated in a real-time fashion with the Illumina Data Collection Software v2.6. After initial base calling, additional custom filtering was performed using calibrated quality scores generated by the Illumina pipeline. Reads generated from both ends of DNA fragments (reads 1 and reads 2) were trimmed by removing from the 3′ ends bases with a PHRED-equivalent quality score below 10. A length threshold of 24 was applied to filtering, indicating that all bases <24 bases in length after trimming were removed from further analysis.

The use of a modified PE PCR primer 2.0 led to a vast majority of read 2 starting with the signature sequence 5′-TGCAGGTGCA-3′. However, in spite of a significant bias in base composition for the first 10 bases of reads 2, a majority of all read 2 data were high quality reads that paired well with their read 1 counterparts. Nonetheless, it must be noted that since Illumina uses images of the first few cycles to calculate phasing, such low 5′ sequence variation could cause base calling errors in subsequent sequencing cycles if a different Illumina instrument or upgraded base calling software were to be used for sequencing. In that particular case, increasing the amount of phiX control DNA spiked into each lane (from ~1% to ~25%) would be recommended. The replacement of *SbfI* (and of its corresponding recognition sequences in the biotinylated *PstI*-specific adapter) by a Type IIs enzyme (such as *BseRI*), thus removing the 10 bp “TGCAGGTGCA” signature sequence from the 5′ ends of reads 2, also is being explored on separate sets of genomic DNA samples.

### 2.4. Alignment to Reference Genome Assembly and *De Novo* Assembly

The 10 bp “TGCAGGTGCA” signature sequence at the 5′ end of read 2 sequences was identified by calculating the Hamming distance with the first 10 bases on read 2. Only read 2 sequences that have a Hamming distance less than or equal to 2 were considered to have a *Pst1* site. Alignment of the reads and contigs to B73 were performed using Bowtie [16]. Local assemblies for each distinct read 2 sequences and their corresponding read 1 sequences were generated using the Velvet software [17] with k-mer values ranging from 23 to 63 with increments of 4 and coverage cutoff values of 4 and 8 for each k-mer value. Of the 22 assemblies generated with varying k-mer and coverage cutoff values, the assembly with the largest contig size was considered the final one for that distinct read 2 sequence and its paired read 1 sequences.

### 2.5. Sanger-Based Validation of Single-Nucleotide Polymorphisms

 Sequences from 100 random B14 and B73 contigs with at least 1 polymorphism to the B73 reference genome v1.0 assembly were used to design PCR primers (length 18–24 bps, Tm 60–65°C) using a local version of the Primer3 primer design software tool. CROSSMATCH analysis was performed on contig sequences using a local maize repeat database to mask repetitive DNA sequences prior to primer design and M13 forward and reverse “tails” were added to the 5′ ends of the PCR primer sequences before ordering. B73 and B14 genomic DNA was subjected to PCR amplification (15 min at 95°C, followed by 40 rounds of 30 s at 95°C, 30 s at 60°C, 1 min at 72°C, and a final extension step of 10 min at 72°C) by using 5 pmol each of the “tailed” PCR primers in a total volume of 10 *μ*L of 1X HotStarTaq Master Mix (Qiagen) containing 25 U HotStarTaq DNA polymerase (Qiagen). 1.5 mmol L^−1^ MgCl_2_ and 200 *μ*mol L^−1^ dNTPs. PCR cleanup reactions were performed by mixing 2 *μ*L of PCR products with 0.75 *μ*L of ExoSAP-IT (USB Corporation) in a total volume of 17 *μ*L with sterile distilled water, and incubating at 37°C for 25 min then 80°C for 25 min. 5 *μ*L of the cleaned-up amplified DNA then were end-sequenced using M13 forward and reverse oligonucleotides and the ABI BigDye version 3.1 Prism sequencing kit. After ethanol-based cleanup, cycle sequencing reaction products were resolved and detected on Life Technologies (Carlsbad, CA, USA) ABI3730xl automated capillary DNA sequences. Individual sequences from each genotype were combined into a single project (one project per amplified fragment) and assembled with the Phred/Phrap/Consed package (see http://www.phrap.org/phredphrapconsed.html). Confirmed SNPs identified on the Genome Analyzer were validated by comparison to single-base mismatches between the B73 and B14 genotypes located in regions that matched the original contig sequence generated by assembling individual read pair sequences with Velvet.

## 3. Results and Discussion

**Figure 1:**
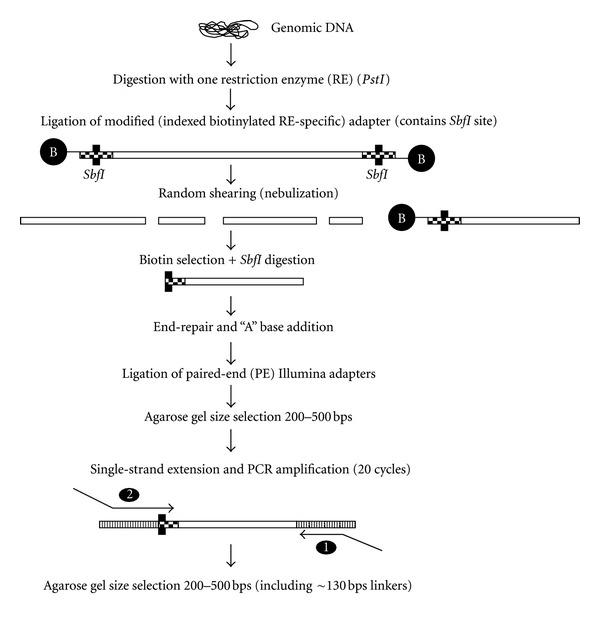
Preparation of paired-end reduced representation libraries. Genomic DNA is digested with methyl-sensitive restriction endonuclease *PstI*. After random shearing, DNA fragments containing the *PstI* end are selected via biotin selection and end-sequenced. Resulting sequences are assembled locally to create large contig sequences.

Library construction and massively parallel sequencing were performed on two public maize inbred lines, B73 and B14, according to the method described in Figure 1. DNA samples first were digested with the methylation-sensitive RE *PstI* (5′-C*TGCA^*∧*^G-3′). The *PstI* activity is blocked by 5-C methylation of the cytosine in the first position (C*) in the recognition sequence. The choice of *PstI* was guided by (1) the intention of enriching for genic regions and avoiding the capture of the repeated fraction of the genome [18, 19], and (2) the potential number of unique sites digested by this enzyme in both samples. 

### 3.1. Illumina Sequencing

 After trimming and filtering, a total of 63.9 million and 94.9 million high-quality read pairs (reads 1 and reads 2) were obtained for B73 and B14, respectively, from one run on the Illumina Genome Analyzer II (Table 1). The disparity between those numbers is due in part to the fact that 3 lanes and 4 lanes of a flow cell were used for B73 and B14, respectively. A total of 61.5 million (96.2% of total) and 92.2 million (97.1% of total) reads 2 for B73 and B14, respectively, contained the signature 5′-TGCAGGTGCA-3′ sequence at their 5′ ends. 

 Since reads 2 are anchored by a methylation-sensitive RE cut site, each read pairs can be grouped based on the identity of their respective read 2 sequences. In order to assign read pairs to specific regions of the genome, and perform local assemblies of such regions, the following analysis was performed: (1) assessing the number of read 2 sequences aligning uniquely to the B73 reference genome assembly, (2) determining the degree of stacking (and the number of distinct reads 2) for all uniquely aligned paired reads 2, and (3) assembling each stacks of distinct read 2 regions covered by more than 100 reads 2 with their corresponding read 1 sequences to generate contigs in targeted regions of the genome. 

### 3.2. Alignment to the Reference Genome Assembly

Only read pairs containing the 10 bp signature 5′-TGCAGGTGCA-3′ sequence at the 5′ end of reads 2 were considered for alignment. After filtering, the 10 bp signature sequence was removed and paired reads 2 were aligned to the B73 reference genome v2.0 assembly using Bowtie, allowing up to 2 mismatches per individual reads. 31.1 million B73 paired reads 2 (50.5% of all paired reads 2 containing the 10 bp signature sequence) and 57.0 million B14 paired reads 2 (61.8%) were aligned uniquely to the B73 reference genome (Table 1). In addition to uniquely aligned reads 2, 13.3 million paired reads 2 in B73 and 22.8 million paired reads 2 in B14 were aligned to multiple regions of the genome, indicating that 72.2% and 86.5% of all paired reads 2 for B73 and B14, respectively, containing the 10 bp signature sequence, align at least once to the B73 reference genome. Finally, a relatively significant fraction of B73 (17 million) and B14 (12.3 million) reads 2 did not align to the B73 reference genome (Table 1). It is possible that those unaligned reads were excluded from the alignment due to the presence of more than 2 mismatches per individual reads that were caused by natural polymorphisms between lines, sequencing errors, or assembly errors in the B73 reference genome v2.0 assembly. Additional BLAST search of a subset of the remaining unaligned B73 and B14 read pairs indicates that at least a fraction of the unaligned sequences contain adapter sequences, suggesting the possibility of DNA fragments ligating to adapters or adapter-adapter ligations during the library construction process.

### 3.3. Genomic Distribution of Distinct Reads 2

**Figure 2:**
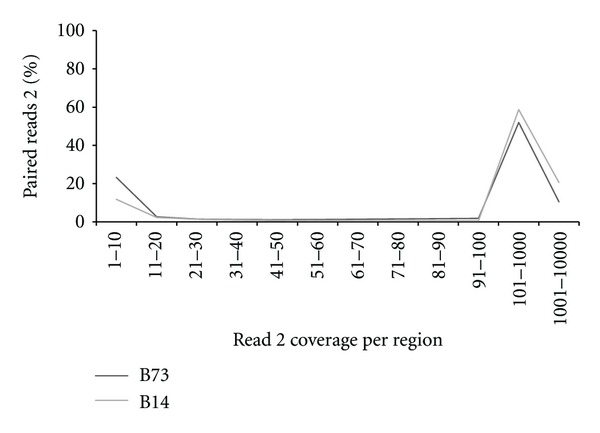
Read 2 coverage of regions by percentages of reads 2 sequences. The percentage of high quality paired reads 2 uniquely aligned to the B73 reference genome is shown in relation to their presence in regions with variable coverage. *Y*-axis: percentage of all high quality reads 2 uniquely aligned to B73 reference genome; *X*-axis: variations in sequencing coverage for regions covered by high quality paired read 2 (e.g., 1–10 = sequencing coverage varying from one read 2 to ten reads 2 for each covered region).

 Sequencing coverage for all uniquely aligned regions of the B73 genome reference v2.0 assembly was assessed by determining distinct read 2 coverage information (Figure 2). Distinct reads 2 (or “regions”) were generated from all uniquely aligned reads 2, gathering all similar and uniquely aligned read 2 sequences into a unique sequence entry. In both lines, 312,924 and 401,379 regions for B73 and B14, respectively, are covered by at least 11 uniquely aligned reads 2 and a majority of uniquely aligned reads 2 (76.7% for B73 and 88.2% for B14) are contained within regions covered by 11 or more reads (and by extension read pairs) (Figure 2), potentially enabling high quality genome-wide *de novo* assembly. The relatively large percentage of distinct reads 2 covered by one to ten reads (1–10 in Figure 2) suggests the presence of sequencing singlets with unchecked sequencing errors and/or the possibility of extraneous contamination during the library construction procedure. Nonetheless, that number is low enough not to decrease the overall quality and usefulness of the analysis. The disparity in number of covered regions between the two lines can be explained at least in part by the higher number of read pairs sequenced for B14 in comparison to B73 and suggest that additional sequencing could further increase the coverage at each individual region adjacent to *PstI* sites in their respective genomes. The number of regions covered by 11 or more reads 2 in B73 in comparison to B14 also suggests that additional sequencing mostly would increase the number of regions covered by 11 reads 2 or more, rather than generating contigs aligning to still unknown regions of interest, although that number also may vary depending on genome content and organization.

### 3.4. Distinct Read 2 Overlap in B73 and B14

 Genomic position overlap between the B73 and B14 distinct read 2 datasets was determined by aligning all distinct reads 2 against the B73 reference genome, using Bowtie and allowing up to two mismatches. The resulting genomic coordinates then were compared between B73 and B14 to assess genomic position overlap. Redundant and nonredundant genomic positions are listed on Table 2 (where distinct reads 2 aligning to more than one region of the genome are considered as redundant). Interestingly, 68.8% and 79.8% of distinct reads 2 in B73 and B14, respectively, and sequenced at least once in one or both genotypes (i.e., sequencing coverage of 1X, or one read 2 sequence) also are sequenced in the other genotype (Table 2). After including reads aligning to multiple regions (up to two mismatches were allowed when aligning distinct reads using Bowtie), 78.2% and 84.9% of distinct reads 2 in B73 and B14, respectively, overlap at the same genomic position. These data suggests that read 2 sequence data, or contigs resulting from assembling paired read 1 and read 2 data, can be used for direct comparison between genotypes for SNP detection and genotyping, in a manner similar to the approach described in Deschamps et al. [8].

### 3.5. *De Novo* Assembly of Individual Read Pairs

**Figure 3:**
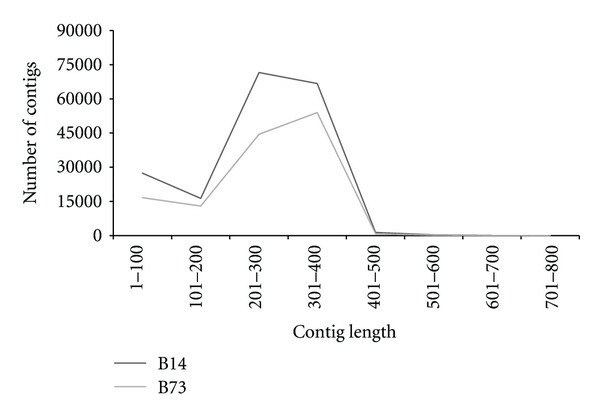
Contig length distribution. The number of contigs generated *de novo* is shown in relation to their length in bps. *Y*-axis: number of contigs generated by assembling paired read 1 and read 2 data extracted from regions with at least 100 stacked read 2 sequences uniquely aligned to the B73 reference genome; *X*-axis: contig length distribution (in bps) (e.g., 1–100 = contigs <100 bps in length).

 Individual read pairs were assembled *de novo* into contigs with the Velvet short read de novo assembler. Only distinct reads 2 generated from uniquely aligned reads 2 and with a sequencing coverage greater than 100 were considered and grouped with their corresponding read 1 sequences for *de novo* assembly. Each group was assembled individually. A total of 183,609 and 129,018 contigs were generated for B14 and B73, respectively. Read usages in both lines as well as the possibility of multiple contigs generated within the same regions likely explain why a higher number of contigs were generated in comparison to the number of regions with a sequencing coverage greater than 100. As suggested by Figure 3, 77% of the B73 contigs (99,431 contigs) and 76.2% of the B14 contigs (139,968 contigs) are >200 bps in length, including several contigs (176 for B73 and 310 for B14) above 500 bps in length, likely artifacts from the gel size-selection process but an indication nonetheless of the potential of the method if larger DNA fragments were to be size-selected on gel.

 Contigs were aligned with Bowtie to the B73 reference genome v2.0 assembly, allowing up to two mismatches. Out of those, 30,763 for B73 and 62,524 for B14 were uniquely mapped to the assembly (Table 3). The lower number of uniquely aligned contigs compared to the total number of contigs shown above may be explained by the fact that most contigs did not extend to the stacked and unique read 2 sequences. As shown on Table 3, Bowtie alignments indicate that a significant fraction of these contigs align perfectly to the assembly (0 mismatch). As expected, a larger fraction of B73 uniquely mapped contigs (72.9%) align perfectly to the assembly, when compared to B14, where only 67.6% align with 0 mismatches. This is due in part to the different genome organization between the two inbred lines and the likely presence of various polymorphisms, including indels in the B14 contigs when compared to the B73 reference assembly. Interestingly, 13.4% of the B73 contigs align to the B73 v2.0 assembly with 1 mismatch and 13.6% align with 2 mismatches, which could be an indication of sequencing or assembly errors in our assembled data or assembly errors in the public data.

 Due to the palindromic nature of *PstI* recognition sites, one valuable side effect of this method is the possibility of generating contigs immediately adjacent to the 5′ end and the 3′ end of the same *PstI* site. The expected outcome would be that such adjacent contigs are in most cases separated by a variable number of bases, as determined by the reference sequence they are aligned against, assuming that contigs are extended from ends opposite the *PstI* ends in DNA fragments assembled together, creating “contig pairs” spanning larger regions of the genome useful for marker development and *de novo * assembly applications. Alignments of contig sequences to the B73 reference genome sequence suggest that 6,495 of the >100 bps uniquely mapped B73 contigs and 28,605 of the >100 bps uniquely mapped B14 contigs generate contig pairs separated by a distance less than 1,000 bps, and centered on a given *PstI* site in their respective genome. In addition, out of those, 680 B73 contigs and 3356 B14 contigs are immediately adjacent contigs centered on a unique *PstI* site. It is expected that deeper sequencing would increase the number of contig pairs per experiment by increasing sequencing coverage and contig length for each discrete genomic regions.

### 3.6. Sequencing Confirmation of Single Nucleotide Polymorphisms

 One application of generating longer contigs from individual read pairs, anchored at a given *PstI* site is the possibility of detecting new SNP in complex genomes and using long contig sequences for primer design and SNP assay development. To determine the feasibility of using contig assemblies for SNP detection, a subset of B73 and B14 contigs with one or more mismatch to the B73 reference genome was selected randomly. Mismatches detected by Bowtie (putative “SNPs”) then were confirmed by Sanger sequencing of PCR products generated using primers derived from their respective contig sequences. A total of 41 amplicons, containing 50 putative SNPs, were sequenced in B14. A total of 45 SNPs were confirmed via Sanger sequencing, while the remaining 5 showed sequencing peaks typical of a heterozygous call, possibly the results of assembly errors in the B73 reference genome of highly conserved regions differing by only one base, assembly errors in the B73 contigs generated by assembling read 2 data from different regions grouped by similar read 1 sequences, or duplication of the corresponding regions in the B14 genome in relation to B73. These data nonetheless confirm that the putative SNPs are present in the DNA fragments of interest, and the quality and value of contig sequences for detecting SNPs in a complex genome-like maize.

 To determine the origin of mismatches in the B73 contig sequences aligned to the B73 reference genome, a similar approach was used, where 40 amplicons, containing 52 putative SNPs, were sequenced via Sanger sequencing of PCR products. Out of the 52 putative SNPs, only 20 were confirmed, while 17 were not confirmed, exhibiting a monomorphic pattern at the base position of interest, and 15 exhibit sequencing peaks characteristic of a heterozygous call. The confirmation of 20 SNPs and the existence of 15 putative SNPs exhibiting sequencing peaks characteristic of a heterozygous call suggests assembly errors in the B73 reference genome or natural variations between DNA materials. The presence of 17 unconfirmed mismatches could be explained by possible sequencing errors. Alternatively, errors in the B73 reference genome assembly could have led to artificial grouping of reads 1 data, led by the fact that uniquely aligned read 2 sequences actually correspond in reality to separate regions of the genomes. A rapid BLAST search of the B73 contig sequences carrying unconfirmed SNPs or SNPs exhibiting sequencing peaks characteristic of a heterozygous call indicates that all or parts of a majority of the contigs (63%, data not shown) align with more than one B73 BAC clone sequence (mapped to different chromosomes), confirming the potential value of filtering such contig sequences prior to SNP discovery or detection. 

## 4. Conclusions

A revised RAD-PE protocol for generating long contig sequences mapping to discrete regions of the maize genome has been tested and implemented. Contig length essentially varies with the size of the DNA fragments selected during the library construction procedure and is expected to be limited by the maximum length of the DNA fragments sequenced on the Illumina platform (separate experiments have shown that DNA fragments up to ~1 Kbps can be sequenced effectively on the Illumina Genome Analyzer). Resulting contigs can be used for several applications, including SNP discovery and marker assay development. They also are expected to facilitate SNP discovery efforts in complex genomes lacking robust reference sequence information, not only in plants but also any other species of interest.

## Figures and Tables

**Table 1:** Run metrics. The numbers of paired reads 2, paired reads 2 containing the 10 bp “TGCAGGTGCA” signature sequence at their 5′ ends, and paired reads 2 aligning to the public B73RefGen_v2 reference genome sequence are indicated.

Run metrics	B73	B14
Number of paired reads 2	63,964,770	94,976,365
Number of paired reads 2 with signature sequence	61,512,151	92,262,878

Alignment against the B73 reference genome sequence^a^		
Align once	31,121,355	57,009,568
Align more than once	13,306,206	22,869,021
Do not align	17,084,590	12,384,289

^a^Best match to reference sequence of reads aligning uniquely or multiple times to the reference sequence with no more than 2 mismatches.

**Table 2:** Position overlap between B73 and B14. The numbers of distinct B73 and B14 reads overlapping at the same genomic position (as determined by the B73_RefGen v2.0 reference genome) are shown, including redundant and nonredundant positions.

	B73	B14
Redundant positions		
Not overlapping	657,961	421,056
Overlapping	2,367,323	

Nonredundant positions		
Not overlapping	731,837	407,130
Overlapping	1,616,620	

**Table 3:** Contig alignment to the B73 reference genome. The number of contigs uniquely aligned to the B73 reference genome assembly and exhibiting 0, 1, or 2 mismatches in relation to the reference are shown.

Number of contigs	B73	B14
0 mismatch	22,436	42,279
1 mismatch	4,142	11,875
2 mismatches	4,185	8,370

Total	30,763	62,524
